# A high-precision, template-assisted, anisotropic wet etching method for fabricating perovskite microstructure arrays†

**DOI:** 10.1039/d0ra07228a

**Published:** 2020-10-16

**Authors:** Xue-fang Hu, Chang-gui Lu, Quan Wang, Jing-kun Xu, Yi-ping Cui

**Affiliations:** Advanced Photonics Center, School of Electronic Science & Engineering, Southeast University Nanjing Jiangsu 210096 China changguilu@seu.edu.cn

## Abstract

Cesium lead-halide (CsPbX_3_; X = Cl, Br, I) perovskite microstructure arrays have become the basis for laser array applications, due to their outstanding spectral coherence, low threshold, and wideband tunability. Furthermore, the common fabrication methods for these arrays have the limitation to achieve both tailored design and high resolution simultaneously. Herein, we report a high-precision, template-assisted, wet etching (TAWE) method for the preparation of perovskite microstructure arrays. This method possesses the advantages of flexible design, controllable size, and ultrahigh accuracy (the resolution can reach 1 μm or higher). A 20 × 20 inverted pyramid array with a diameter of 3 μm and a period of 4 μm was fabricated using this method. CsPbBr_3_ perovskite quantum dots fabricated by means of hot injection were filled into the inverted pyramid array *via* spin-coating and pumped using a laser with a wavelength of 400 nm. The lasing characteristics of the array were then measured and analyzed; the threshold was measured to be 37.6 μJ cm^−2^, and the full width at half maximum of the amplified spontaneous emission spectrum was found to be about 4.7 nm. These results demonstrate that perovskite microstructure arrays prepared *via* this method have potential applications in laser arrays.

## Abstract 2

A template-assisted wet etching method for the preparation of perovskite micro-structure array is proposed. This method has a superiority of flexible graph design, controllable size and high precision.

## Graphical abstract

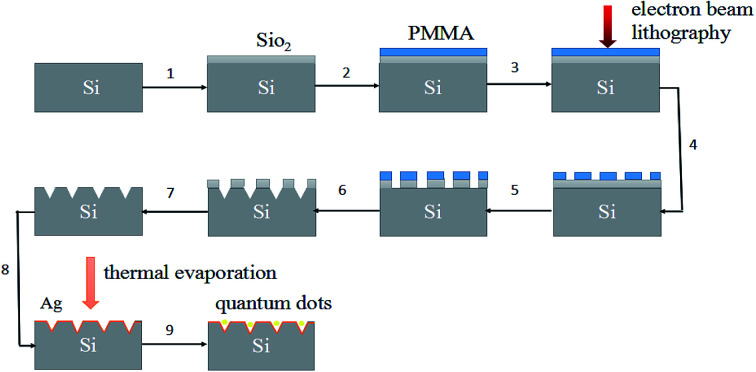

## 1 Introduction

All-inorganic cesium lead-halide (CsPbX_3_; X = Cl, Br, I) perovskites are deemed to be ideal materials for the optical gain medium in optoelectronic devices, because of their superior optical properties such as high photoluminescence quantum yields (PLQYs),^1,2^ narrow emission line width,^3^ wide emission spectra tunability,^4^ and low material cost.^5^ These properties make them a prime candidate for use in the field of solar cells,^6^ white light-emitting diodes,^7^ displays,^8^ photodetectors and lasers in particular.^9,10^ The lasing behaviors of perovskites are strongly dependent on the quality of the crystal and the physical structure. Since the discovery of amplified spontaneous emission (ASE) and laser emission in perovskite films, numerous laser devices with high quality factors (*Q*-factors), low thresholds and optimum dimensions have been demonstrated for perovskite-based microstructural materials, including nanowires,^11,12^ microplates,^13–15^ cube-corner pyramids^16^ and hexagonal platelets.^17,18^ It is unavoidable that modern photoelectric devices will become integrated and arrayed, benefiting and expanding their practical applications. This makes the fabrication of perovskite microstructure arrays significant.

There are many ways to fabricate a perovskite microstructure array. The most common methods are the template method, direct writing and the vapor phase method. The template method provides a facile, highly efficient and refined preparation strategy. Mass production is also possible using the template method, but it is insufficient in the flexibility of design and alignment, meaning that this method cannot fully meet the requirements for developing perovskite array devices.^19^ Although the direct writing method shows superiority in its flexibility of design, low cost and efficient mass production, the resolution of the existing direct writing method is still relatively low.^20^ As for the vapor phase method, crystal defects are few, but it generally possesses disadvantages such as high temperature, poor controllability and expensive equipment.^21^ Therefore, it is urgent to develop new preparation methods for perovskite microstructure arrays.

In this paper, we propose a template-assisted wet etching (TAWE) method for the preparation of perovskite microstructure arrays. This method possesses the superior advantages of flexible design, controllable size and high precision (the resolution can reach 1 μm or higher).^22^ Since the laser array properties are closely related to the size and shape of the microstructure, this method could provide a convenient way to optimize the laser characteristics through changing the design parameters of the microstructure arbitrarily. A 20 × 20 inverted pyramid structure array was fabricated using this method to verify its feasibility. CsPbBr_3_ perovskite quantum dots fabricated *via* hot injection were filled into the inverted pyramid array by spin-coating and pumped using a laser with a wavelength of 400 nm. The lasing characteristics were measured and analyzed; the threshold was found to be 37.6 μJ cm^−2^, and the full width at half maximum (FWHM) of the amplified spontaneous emission spectrum was about 4.7 nm. The experimental results demonstrate that the microstructure array prepared using this method has potential applications as a laser array.^23,24^

## 2 Experimental principles and microstructure preparation

### 2.1 Anisotropic wet etching

**Fig. 1:**
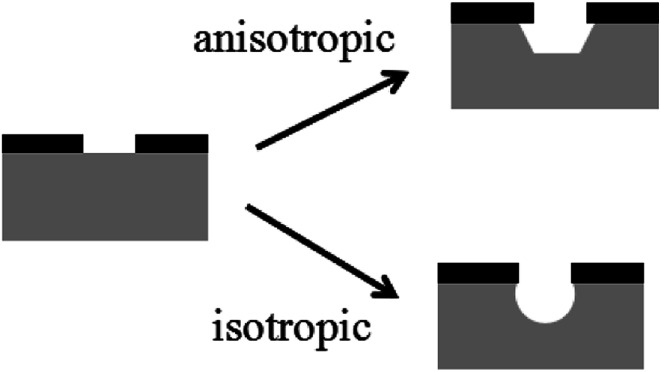
A comparison between anisotropic and isotropic wet etching.

A comparison of anisotropic and isotropic etching is depicted in Fig. 1. Isotropic wet etching proceeds at the same speed for each crystal orientation during the etching process, while for anisotropic wet etching, the speed is higher for certain crystal orientations. Anisotropic wet etching is the foundation of the TAWE method to prepare perovskite microstructure arrays.

### 2.2 The TAWE method

**Fig. 2:**
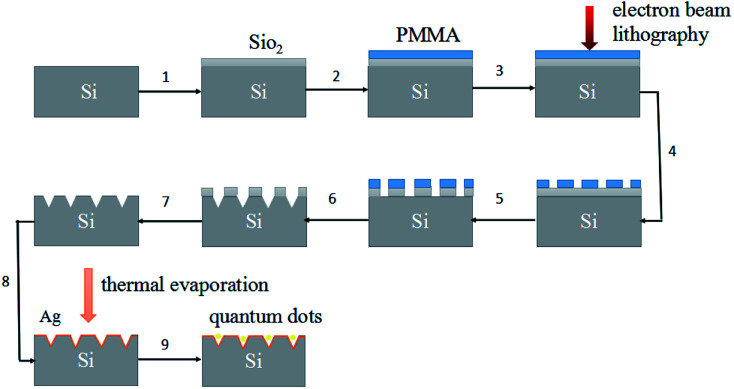
A diagram illustrating the TAWE method.

Fig. 2 illustrates the typical process of the TAWE method, which includes three main steps: (1) preparation of template using electron beam lithography technology; (2) etching of the template *via* isotropic wet etching and coating with a silver film through thermal evaporation; (3) fabrication of CsPbBr_3_ inorganic perovskite quantum dots *via* hot injection and filling them into the inverted pyramid array through spin-coating (see details in ESI, Fig. S1†).

### 2.3 Preparation of CsPbBr_3_ quantum dots

**Fig. 3:**
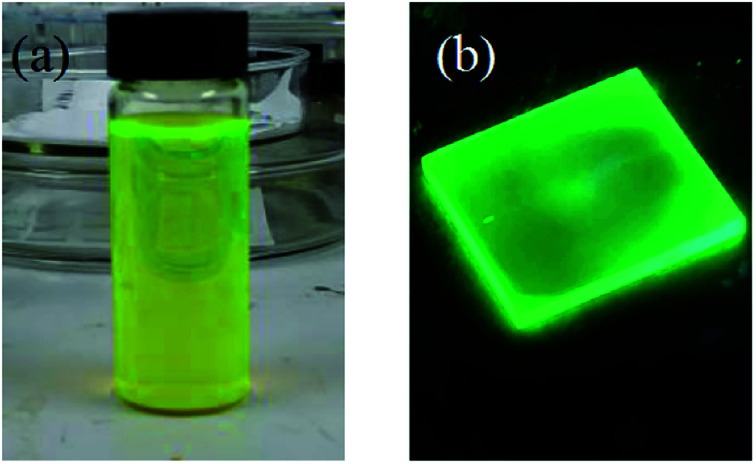
(a) The CsPbBr_3_ perovskite QD solution. (b) Light emission.

There are many ways to fabricate the CsPbBr_3_ quantum dots (QDs), including hot injection,^25,26^ ion exchange^27^ and chemical vapor deposition.^28^ In this paper, the perovskite QDs were synthesized by the hot injection method, which is widely used. It mainly consists of two steps: (1) synthesis of a cesium oleate precursor; (2) heating and dissolving lead bromide in organic solvent, and then injecting the cesium oleate precursor into the lead bromide solution under a nitrogen atmosphere. The perovskite QDs solution was obtained after cooling and dispersing (see details in the ESI†). Fig. 3a shows a picture of the synthesized CsPbBr_3_ perovskite QDs solution, which presented a yellow color, and uniformly dispersed QDs. Fig. 3b shows light emission after spinning the QDs on a glass substrate and illuminating with ultraviolet light. The CsPbBr_3_ perovskite QDs present good emission properties.

## 3 Structural characterization

**Fig. 4:**
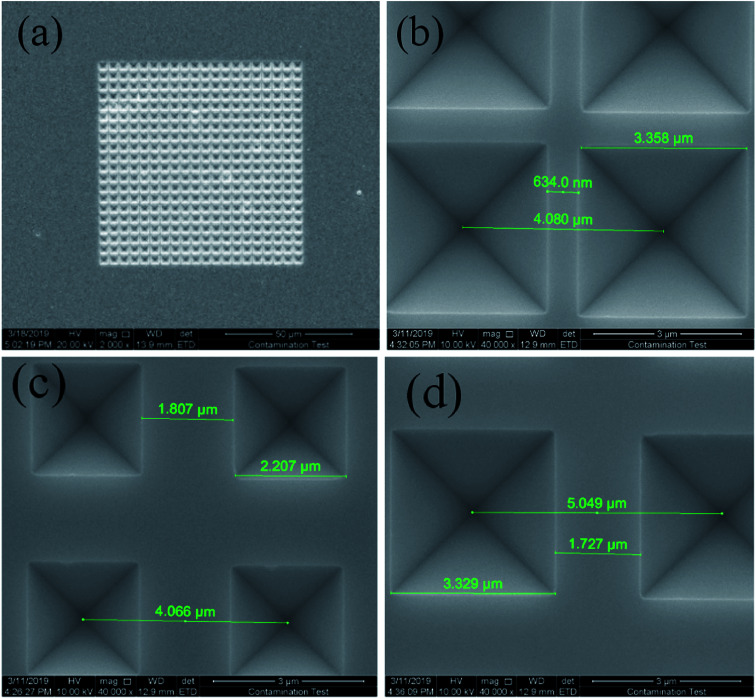
SEM images of the inverted pyramid arrays: (a) integral; (b), (c), and (d) partial SEM images of S_A_, S_B_, and S_C_.

Fig. 4 presents a scanning electron microscope (SEM) image of the inverted pyramid microstructure array. Fig. 4a is the integral view of the inverted pyramid microstructure array with a size of 20 × 20 (80 μm × 80 μm). This image demonstrates that every inverted pyramid is complete and distributed uniformly. Fig. 4b (sample S_A_) shows a partial view of the inverted pyramids with a diameter of 3 μm and a period of 4 μm. For comparison, inverted pyramid arrays with pyramid diameters of 2 μm and 3 μm, and corresponding periods of 4 μm and 5 μm, respectively, are shown in Fig. 4c (sample S_B_) and d (sample S_C_). These results demonstrate the feasibility of the TAWE method in accurately fabricating microstructure array templates with different sizes and periods. The resolution can reach 1 μm or higher, depending on the precision of the electron beam lithography technology. Since the lasing properties of the laser array are closely related to the physical structure, the TAWE method could provide a convenient way to optimize the laser characteristics through arbitrarily changing the design parameters of the microstructure. Therefore, we compared the differences in the ASE threshold between S_A_, S_B_ and S_C_. The results show that the larger sized inverted pyramid template has a lower ASE threshold, which verifies the significance of the architecture to the lasing properties (see ESI, Table S1†).

**Fig. 5:**
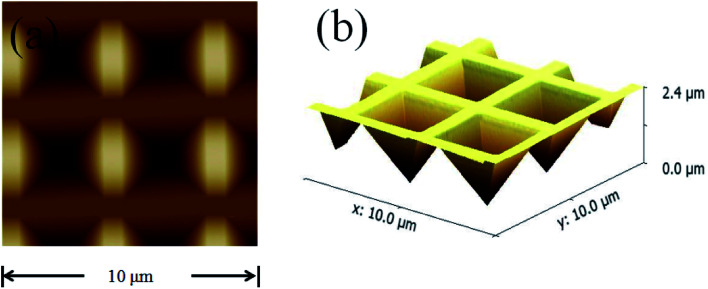
Atomic force microscope images of the inverted pyramid array: (a) top view; (b) three-dimensional view.

In the measurement of laser characteristics, the perovskite QDs were filled into the inverted pyramid array to act as a gain medium, while the inverted pyramid microstructure played the role of a cavity.^24,25^ A silver film with a thickness of 250 nm was deposited on the structure to reduce cavity dispersion, radiation loss and threshold loss. However, considering that the silver film could easily fall off the silicon wafer, a chromium film with a thickness of 10 nm was deposited as an adhesion layer. In order to ensure the quality of the silver film, it was necessary to heat to 150 °C during evaporation. Fig. 5 shows an atomic force microscope (AFM) image of the fabricated inverted pyramid array. Fig. 5a is the top view of the sample, and Fig. 5b is the three-dimensional view of the sample, with a depth of 2.4 μm.

**Fig. 6:**
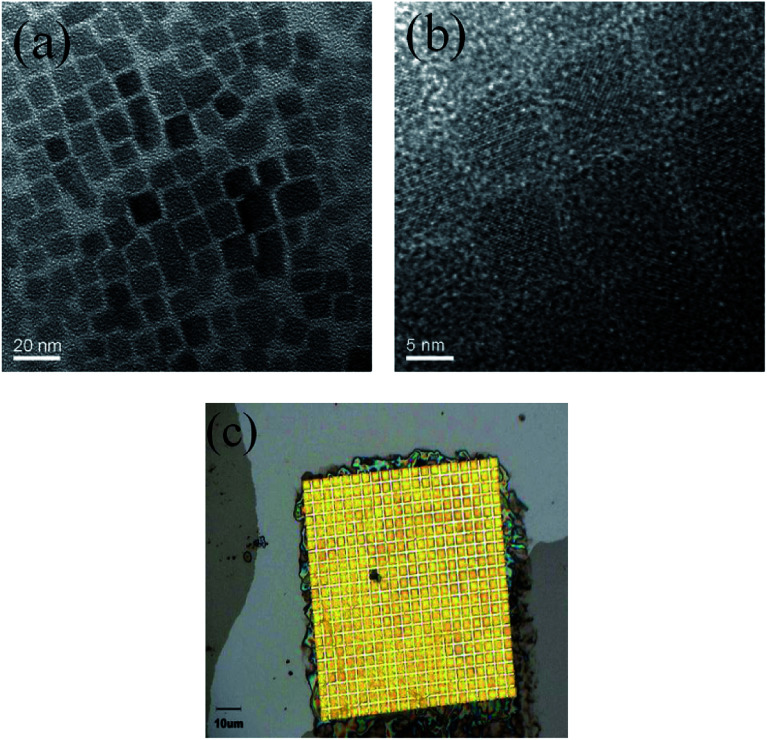
Transmission electron microscope images of CsPbBr_3_ perovskite QDs at (a) low and (b) high resolution. (c) An optical microscope image of the inverted pyramid array filled with perovskite.

Transmission electron microscopy (TEM) images of the CsPbBr_3_ QDs are shown in Fig. 6a and b, revealing QDs with an average size of 10 nm. We also measured the sample using an optical microscope to confirm whether the QDs were completely filled into the inverted pyramid structure. As shown in Fig. 6c, almost all inverted pyramid structures are uniformly filled with perovskite QDs. The perovskite QDs at the edge of the structure were removed by tape, and a more compact CsPbBr_3_ perovskite film could be obtained by cycling spin-coating and drying several times.

## 4 Optical characteristics

**Fig. 7:**
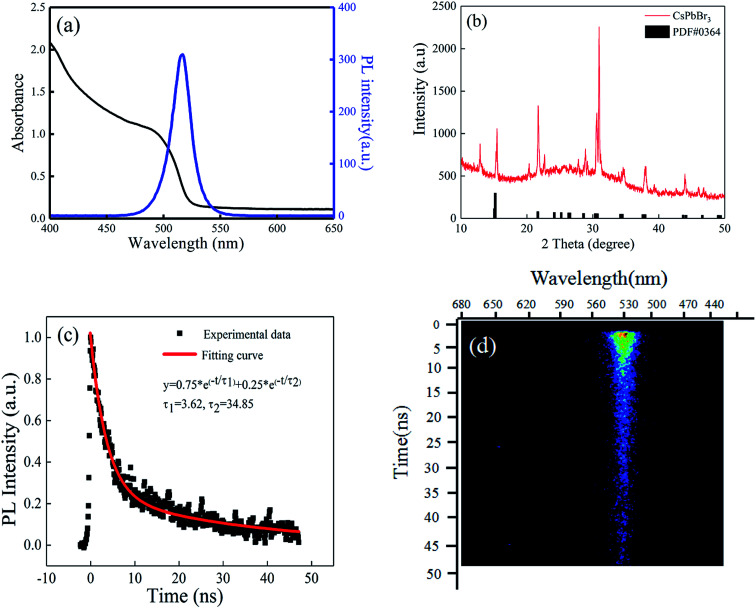
(a) Photoluminescence and absorbance spectra of the CsPbBr_3_ QDs solution. (b) The XRD pattern of the CsPbBr_3_ film. (c) Time resolution of the PL spectrum of CsPbBr_3_ QDs solution. (d) PL decay of the CsPbBr_3_ QDs solution.

The absorption and photoluminescence (PL) spectra of the CsPbBr_3_ perovskite QDs solution are depicted in Fig. 7a. The PL spectrum displays an obvious peak at 529 nm, which is the emission peak of the perovskite QDs, and the FWHM of the spectrum is about 20 nm. X-ray diffraction (XRD) of the CsPbBr_3_ perovskite film (prepared through spin-coating the QDs solution on a glass base) was recorded and is shown in Fig. 7b. The strong intensity of the diffraction peaks indicates the good crystallinity of the film. Most of the peaks are in agreement with standard (rhombic) CsPbBr_3_ (PDF#18-0364). We also measured the emission lifetime using a streak camera (c5680-04/M, Hamamatsu). Fig. 7c shows the PL lifetime of the CsPbBr_3_ QDs solution. The PL lifetime of the CsPbBr_3_ QDs solution is 3.62 ns and 34.85 ns, which is fitted by a double-exponential function. The PL decay of the CsPbBr_3_ QDs solution is also depicted in Fig. 7d.

**Fig. 8:**
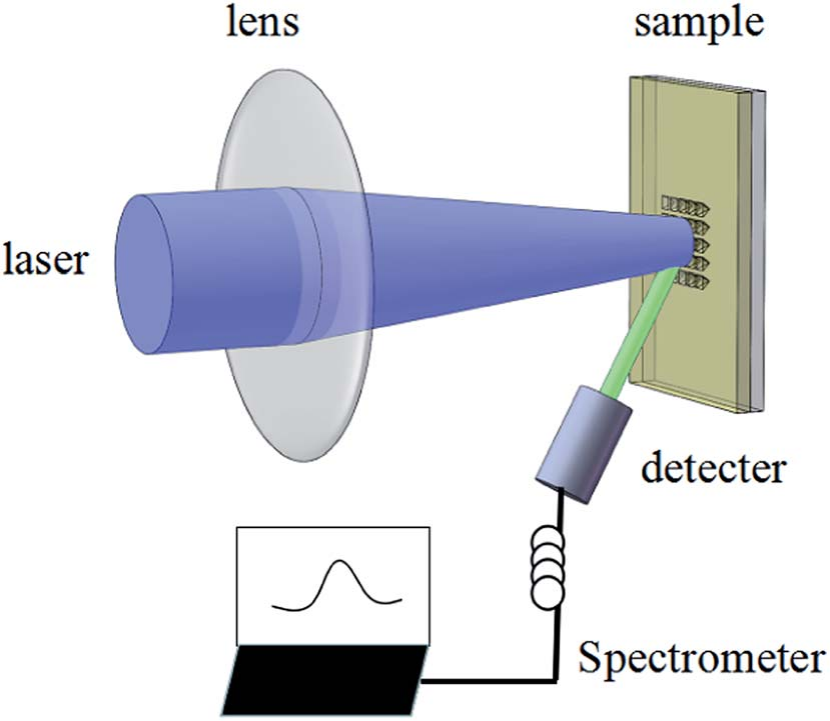
A schematic diagram of the experimental light path.

A schematic diagram for the ASE measurement is illustrated in Fig. 8. The excitation source used for this work was a Ti:sapphire oscillator/amplifier. The latter produced ∼120 fs duration, 800 nm wavelength laser output with a repetition rate of 1 kHz. The output divergence angle after collimation was around 0.65 mrad. By using a beta barium borate (BBO) crystal, the output wavelength was converted to 400 nm. The pump beam was focused by a lens on the sample, while the ASE signal was collected by the spectrograph.

**Fig. 9:**
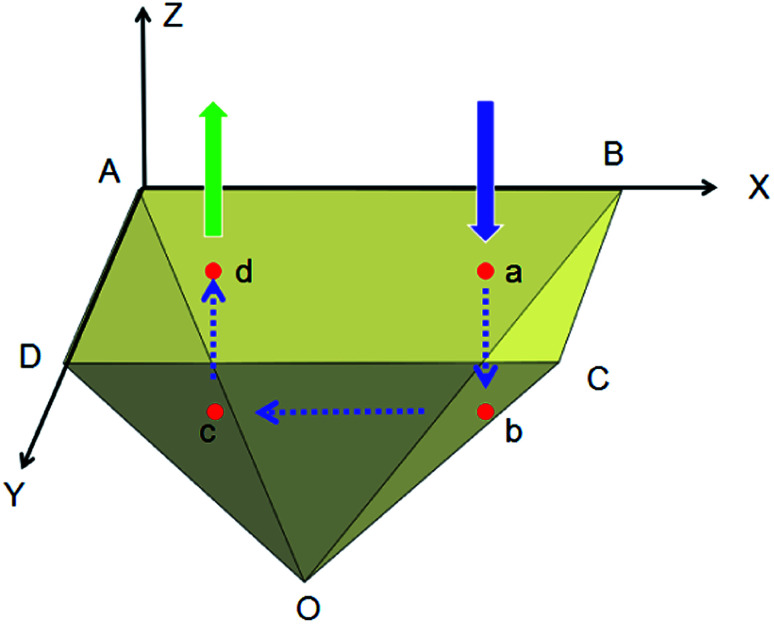
The optical path in the inverted pyramid.

The reflected light path inside the inverted pyramid structure is shown in Fig. 9. The confined light travels along the blue-line path of a–b–c–d (a, d in plane ABCD, b in plane OBC, c in plane OAD), emitted from point d on plane ABCD and amplified, which can eventually be detected by the spectrometer. Emission is also possible through a different path or other reflections in the inverted pyramid structure, leading to a multimode.

**Fig. 10:**
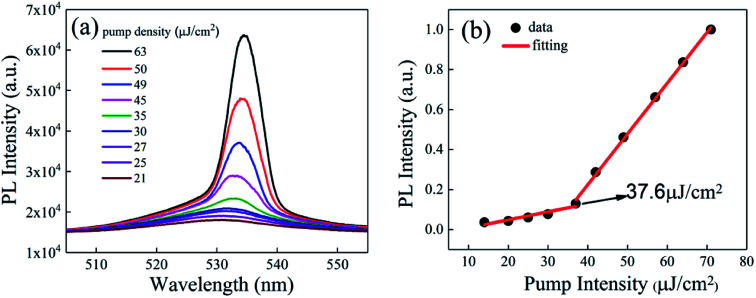
(a) Photoluminescence spectra of the inverted pyramid perovskite array. (b) Photoluminescence intensity *versus* pump intensity.

The laser characteristics of the inverted pyramid perovskite microstructure array were measured using the experimental light path depicted in Fig. 8. The intensity of the laser light incident on the sample changed slowly by adjusting the attenuator, and the spectrum of the sample was recorded, as shown in Fig. 10a. Its photoluminescence (PL) intensity *versus* the pump intensity is shown in Fig. 10b. The sample under low pump power presented a broad spontaneous emission (SE). With increasing pump density, a peak at 536 nm emerged and quickly became dominant. Meanwhile, the FWHM of the emission spectrum narrowed sharply, which signified the transition from SE into an ASE regime.^29–31^ Thus, the threshold is 37.6 J cm^−2^, and the FWHM is about 4.7 nm, revealing that the inverted pyramid perovskite microstructure array presents good laser characteristics.^32^ A better PL stability could be obtained when the inverted pyramid perovskite array was stored under vacuum or N_2_ atmosphere, instead of being exposed to air. The luminescence stability and photostability were also measured (see ESI, Fig. S2†).

## 5 Conclusions

In conclusion, we reported a high-precision, template-assisted, wet etching (TAWE) method for the preparation of perovskite microstructure arrays. This method shows obvious superior advantages in relation to design, size controllability, and precision. A 20 × 20 inverted pyramid array with a diameter of 3 μm and a period of 4 μm was obtained using this method. The laser characteristics of the structure were tested and analyzed, and the threshold was found to be 37.6 μJ cm^−2^, and the FHWM was 4.7 nm. The results demonstrate that the microstructure array prepared using this method has potential applications in laser arrays.

## Conflicts of interest

There are no conflicts to declare.

## Supplementary Material

RA-010-D0RA07228A-s001
