# Supplementary material for: A high-precision, template-assisted, anisotropic wet etching method for fabricating perovskite microstructure arrays
Source: RSC Adv. 2020 Oct 16;10(63):38220–6. doi: 10.1039/d0ra07228a (PMC9057178; doi:10.1039/d0ra07228a)
Supplement: RA-010-D0RA07228A-s001 [file RA-010-D0RA07228A-s001.pdf]

Electronic Supplementary Material (ESI) for RSC Advances.
This journal is © The Royal Society of Chemistry 2020

A High-precision Template Assisted Anisotropic Wet Etching Method for Perovskite Micro-structure Array

Xue-fang Hu ,Chang-gui Lu *, Quan Wang, Jing-kun Xu , Yi-ping Cui

Advanced Photonics Center, School of Electronic Science & Engineering, Southeast University, Nanjing, Jiangsu,210096, China

**Corresponding author: changguilu@seu.edu.cn*

1 Experimental Section

1.1. Chemicals. All chemical reagents were of analytical grade and were used as received without further purification. oleic acid(OA), oleylamine (OAm, 80-90%), lead bromide ($PbBr_2$, 98.00%) and cesium bromide (Cs_2CO_3, 99.99%) were purchased from Aladdin. toluene were purchased from Sinopharm Chemical Reagent Company of china. octadecene ($C_{18}H_{36}$, 90%) were purchased from Aldrich. ethyl acetate($C_3H_6O_2$, 98%) were purchased from Aesar.

1.2. Preparation of the $CsPbBr_3$ quantum dots. (1) the Cs_2CO_3 (0.8 g) were dissolved into the bottle mixed with the Octadecene (30ml) and the oleic acid(2.5ml), the Cesium oleate precursor was obtained until the reaction completely finished at a temperature of 150℃under the N_2 atmosphere (2) Mix the Octadecene (10ml), oleic acid(1ml), oleylamine (1mL) and lead bromide (0.36 mmol) in the bottle and heat to 180℃until the lead bromide dissolve completely at the N_2 atmosphere, and add the prefabricated Cesium oleate precursor (0.8ml) into the mixed solution. (3) added some toluene and ethyl acetate into the mixed solution, take out the crystal precipitation at the bottom and redispersing in toluene, the $CsPbBr_3$ quantum dots is fabricated.

1.3. ASE and lasing Measurements. For the ASE measurements, all experiments were conducted at room temperature. The excitation source used for this work was a Ti: sapphire oscillator/amplifier. The latter produced ~120 fs duration, 800 nm wavelength laser output with a repetition rate of 1 kHz. The output divergence angle after collimation was around 0.65 mrad. By using a beta barium borate (BBO) crystal, the output wavelength was converted to 400nm. The pump beam was focused by a lens on the sample, and the emission from the edge of the structure was collected by a spectrograph (spectrapro-300i, Acton research corporation).

1.4 Device Measurements. The electron beam lithography technology is using the equipment (Inspect F50, FEI).Transmission electron microscopy (TEM) images were taken with an accelerating voltage of 200 kV (Tecnai G20, FEI).The atomic

force microscope (AFM) were recorded using a microscope (Bioscope Resolve, Bruker).Scanning electron microscopy (SEM) images were taken with an accelerating voltage of 20 kV (Inspect F50, FEI). The absorption and PL intensity spectra were measured through a spectrophotometer (UV-3600, SHIMADZU) and spectrofluorophotometer (RF-5301PC, SHIMADZU) respectively.

2.2 The preparation of the pyramid array structure

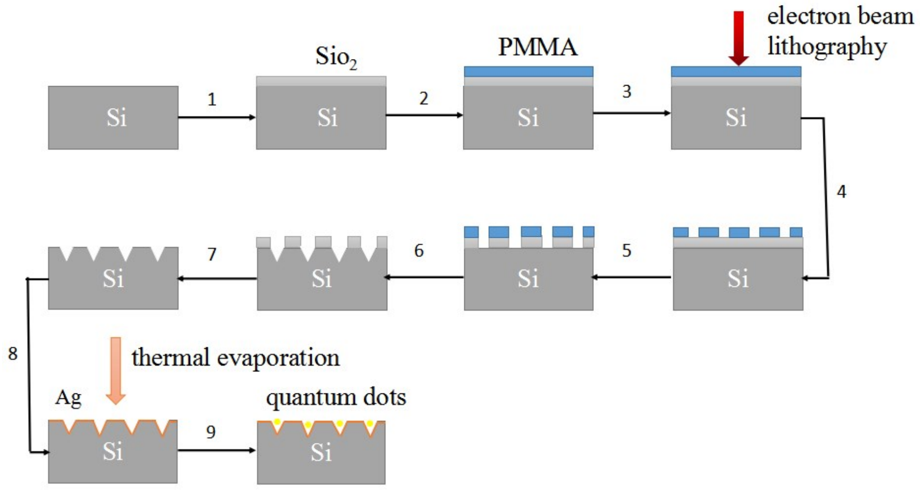

Fig. S1 The diagram of TAWE method

The template assisted wet etching (TAWE) method main contains following steps:

(1) A silica with a thickness of 30 nm was deposited on the silicon substrate through chemical vapor deposition.

(2) A PolyMethylMethacrylate (PMMA) photoresist with a thickness of 200 nm was spun on the sillica through spin coating.

(3) The graph designed in computer was transferred to the PMMA through the electron beam lithography technology.

(4) Mask structure was fabricated after development process.

(5) The sample was put into the HF solution to transfer the mask structure from PMMA to the sillica.

(6) Anisotropic wet etching of silicon was processed using an alkaline solution, and then, the PMMA was removed by the acetone.

(7) The sillica was removed by the HF solution.

(8) The Ag film with a thickness of 250 nm was coated through the thermal evaporation technology. A chromium film with a thickness of 10 nm is coated act as a adhesion layer. In order to ensure the quality of the silver film, it was necessary to keep at an appropriate temperature (150℃) during evaporation.

(9) The $CsPbBr_3$ quantum dots fabricated through hot injection is filled into the inverted pyramid micro-structure array through spin coating method, and a smooth $CsPbBr_3$ perovskite film can be formed in the micro-structure by cycling spin-coating

and dry several times.

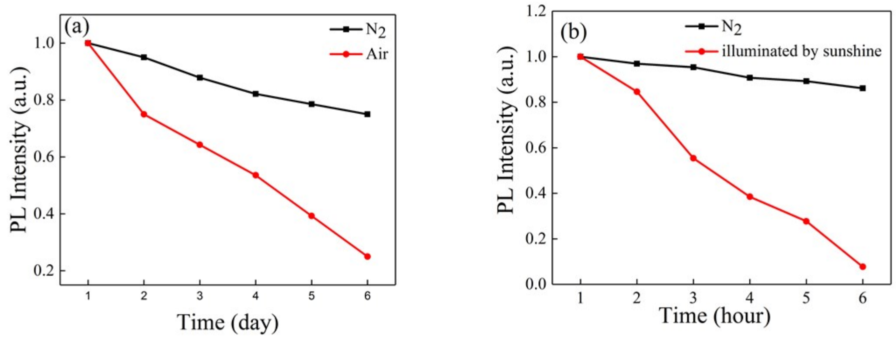

Fig. S2 (a)The stability of the PL intensity versus the time (b)The stability of the PL intensity versus the exposure time

Table S1 The ASE threshold of the sample S_A, S_B and S_C

The sample	S_A	S_B	S_C
Threshold($\mu J/cm^2$)	37.6	54.1	39.5
